# Transarterial Chemoembolization Combined With Hepatectomy for the Treatment of Intermediate-Stage Hepatocellular Carcinoma

**DOI:** 10.3389/fonc.2020.578763

**Published:** 2020-11-04

**Authors:** Qunfang Zhou, Fei Tuo, Ruixia Li, Xiaohui Wang, Juncheng Wang, Zhimei Huang, Minshan Chen, Jinhua Huang

**Affiliations:** ^1^ Department of Minimally Invasive Interventional Therapy, Sun Yat-sen University Cancer Center, Guangzhou, China; ^2^ Department of Ultrasound Diagnose, The First Hospital of Hunan University of Chinese Medicine, Changsha, China; ^3^ Department of Hepatobiliary Oncology, Sun Yat-sen University Cancer Center, Sun Yat-sen University, Guangzhou, China

**Keywords:** combination therapy, hepatectomy, intermediate hepatocellular carcinoma, overall survival, transarterial chemoembolization

## Abstract

**Background:**

Transarterial chemoembolization (TACE) is currently the recommended treatment for intermediate-stage hepatocellular carcinoma (HCC). Liver resection (LR) may be an effective option, although recurrences are not uncommon. TACE prior to LR has been proposed as an even better alternative.

**Methods:**

Patients with intermediate-stage HCC who underwent curative resection were enrolled between January 2007 and December 2015. We compared overall survival (OS) and recurrence-free survival (RFS) for the 2 groups using the Kaplan-Meier method, and we determined independent risk factors for death and recurrence using multivariate regression analyses.

**Results:**

A total of 488 patients with HCC at BCLC B (265 patients with LR, 223 patients with TACE+LR) enrolled from our center. Mean follow-up was 40.2 (range, 3.0–128.7) months. For patients receiving TACE+LR and LR, estimated 1-, 3-, and 5-year OS rates were 90.6% and 73.3%, 61.7% and 43.5%, and 52.9% and 33.8%, respectively (all *P* < 0.001) and estimated 1-, 2-, and 3-year RFS rates were 54.6% and 39.4%, 41.4% and 29.4%, and 36.3% and 26.3%, respectively (*P* < 0.001, *P* = 0.002, and *P* = 0.008, respectively). Significant independent predictors of poor OS were more than 3 (vs. 3 or fewer) tumors (HR=2.19, 95% CI 1.69–2.84), non-anatomical (vs. anatomical) hepatectomy (HR=1.29, 95% CI 1.01–1.66), microscopic vascular invasion (HR=1.46, 95% CI 1.15–.90), cirrhosis (HR=2.41, 95%CI 1.88–3.01), and intraoperative blood transfusion (HR=1.29, 95% CI 1.01–1.66).

**Conclusion:**

Preoperative TACE with LR may result in better oncological outcomes than either TACE or LR alone, without a substantial increase in morbidity, and could be considered an effective combination treatment for intermediate-stage HCC.

## Introduction

Hepatocellular carcinoma (HCC) is one of the most common cancers in the world, ranked as the sixth most common neoplasm and the third leading cause of cancer-related mortality worldwide (1). Advances in diagnostic imaging and widespread application of screening programs in high-risk populations have allowed detection of HCC at earlier stages, but some patients with HCC still continue to present in intermediate or even advanced stages. According to the Barcelona Clinic Liver Cancer (BCLC) algorithm for the treatment of HCC, intermediate-stage HCC (stage B) is defined as extensive multifocal disease without vascular invasion in patients with preserved liver function and the absence of cancer-related symptoms (2).

However, intermediate-stage HCC actually involves a heterogeneous group of patients, encompassing those with a wide range of tumor sizes (larger than 3 cm to over 10 cm) and numbers (2 to over 20), provided that patients have good liver function (Child-Pugh classes A or B) (3). Likewise, the prognosis of patients with intermediate-stage HCC varies (4). According to the BCLC algorithm, transarterial chemoembolization (TACE) is the recommended treatment for patients with intermediate-stage HCC (5). Whereas TACE has been reported to extend the survival of groups of patients with intermediate-stage HCC, the outcomes for individual patients treated with TACE for intermediate-stage HCC have remained mixed (6). At the present time, it remains controversial whether there is enough evidence supporting TACE, particularly relative to liver resection (LR), as the best treatment for patients with intermediate-stage HCC (7).

In fact, multiple recent reports have suggested that LR, when compared to TACE, might provide a survival benefit to patients with intermediate-stage HCC (8–10). Historically, LR has been reserved for the treatment of patients with early−stage HCC who have good liver function (11). Yet with improvements in surgical techniques and perioperative care, the surgical mortality rate for LR in patients with HCC has been reduced to less than 1% (12, 13). In addition, the complete surgical removal of the tumor may offer the best chance for long-term survival in patients with HCC. Nevertheless, patients who have LR for HCC larger than 5 cm often relapse after a short recurrence-free interval, especially those patients with huge (10 cm or larger) HCC (14, 15).

At the same time, TACE has been used successfully as a neoadjuvant therapy for large HCC prior to LR (16). A 2018 systematic review demonstrated that TACE can feasibly be combined with other modalities to improve the resectability rate for HCC (17). Along these lines, the use of preoperative TACE followed by LR has been shown to improve survival outcomes for some patients with large HCC (18).

Over the years, our Cancer Center has treated a relatively large population of patients with intermediate-stage HCC. Provided that liver function reserve was adequate and complete resection of the tumor appeared feasible, we offered LR to these patients. For some, we also recommended preoperative TACE, with the belief that this might potentially reduce postoperative recurrences and improve long-term survival. Our hypothesis has been that some patients with BCLC stage B may benefit from not only LR but also preoperative TACE. The aim of this retrospective study was to identify patients at our Cancer Center with intermediate-stage HCC who had LR and others who had TACE prior to LR, to compare the outcomes of each approach using survival rates, and to determine the prognostic factors for recurrence and death in these patients.

## Methods

This study was conducted in accordance with the principles of the Declaration of Helsinki (19), and the study protocol was approved by the Ethics Committee of the Sun Yat-sen University Cancer Center.

### Study Population

We retrospectively reviewed the medical records of patients who received a diagnosis of HCC from January 2007 to December 2015 at our Cancer Center. The diagnosis of HCC was made using criteria defined by the American Association for the Study of Liver Disease and the European Association for the Study of the Liver, and was based either on positive liver biopsy or characteristic findings on imaging (multiphasic CT or dynamic contrast-enhanced MRI) combined with serum Alpha-fetoprotein (AFP) levels (20, 21). The clinical stage of HCC was determined according to the BCLC guidelines (22).

The inclusion criteria for this study included: (a) age 18 to 75 years; (b) Eastern Cooperative Oncology Group (ECOG) performance status score of 0 or 1; (c) HCC with 2 or more tumors, at least one of which with a diameter greater than 3 cm, confirmed on postoperative pathological examination; (d) no macrovascular invasion or extrahepatic metastasis; (e) adequate liver function (i.e., Child–Pugh class A or B liver function); (f) adequate renal function (i.e., serum creatinine concentration no higher than 1.5 times the upper limit of normal); and (g) adequate coagulation function (i.e., prothrombin activity > 40%, international normalized ratio [INR] < 1.26, and platelet count > 50 × 10^9^/L). Patients were excluded from the analysis for any of the following reasons: (1) under 18 years or over 75 years of age; (2) recurrent HCC; (3) only a single HCC tumor of any size, or multiple HCC tumors but all with diameters of 3 cm or less; (4) received previous systematic chemotherapy, targeted (Sorafenib) therapy, or radiofrequency ablation (RFA) for HCC; (5) lost to follow-up within 90 days after LR; or (6) information about prognostic variables or follow-up could not be obtained.

### Demographic and Clinicopathological Characteristics

We collected data about each patient’s demographic and clinical characteristics, including sex, age, body mass index (BMI), Child-Pugh grade (severity of liver disease, based on 5 clinical factors: PT or INR, albumin, bilirubin, ascites, and hepatic encephalopathy), diameter of largest HCC tumor, number of tumors, preoperative hepatitis (based on history of chronic HBV infection and/or positive hepatitis B virus RNA testing), preoperative portal hypertension (defined as esophageal varices and/or splenomegaly on imaging studies, combined with a decreased platelet count [100 × 10^3^/μL or less]), and preoperative blood testing (including AFP, liver and renal function tests, prothrombin time [PT] and international normalized ratio [INR], and complete blood count).

We also collected data for each patient about their histopathological findings from LR (microvascular invasion and cirrhosis [of the noncancerous part of the resected specimen]), volumes of intraoperative blood loss and intraoperative blood transfusion, and postoperative complications (large pleural effusion, pneumonia, portal vein thrombosis, cholestasis, and/or ascites).

### TACE Procedure

The decision to utilize TACE before LR was made by the treating physician and was based on the patient’s liver function as well as the number, size, and degree of enhancement of HCC tumors observed in imaging studies. Patients receiving TACE had it administered within 3 months of LR. TACE was carried out under the guidance of digital subtraction angiography (DSA) (Allura Xper FD 20, Philips), and it was performed through the left or right hepatic artery, and directly through a tumor-feeding artery when technically possible. Hepatic artery angiography, which was performed using a 5 Fr (RH or Yashiro) catheter, was first used to assess the location, number, size, and blood supply of the target tumors. The embolization emulsion was a mixture of Epirubicin (Farmorubicin; Pharmacia, Tokyo, Japan) 30 mg to 60 mg, Lobaplatin (Chang’an International Pharmaceutical, Hainan, China) 30 mg to 50 mg, and Lipiodol (Laboratorie Guerbet, Aulnay-sous-Bois, France) 10 mL to 30 mL, and it was infused into tumor-feeding arteries *via* a 2.7/2.8 Fr micro-catheter. The doses of the agents contained in the embolization emulsion were selected based on patient age, weight, comorbidity, tumor size, tumor number, and anticipated tolerance. The endpoint of the TACE procedure was reached when there was no flow in the tumor-feeding vessels.

### Liver Resection

Liver resection was performed by experienced surgeons. We developed a surgical plan based on tumor size, tumor location, and liver function. The hepatectomy method contains anatomical resection and non-anatomical resection, and the extent was defined using the Brisbane 2000 Terminology of Liver Anatomy and Resections (23). We applied Pringle’s maneuver with cycles of clamping and unclamping times of 1 to 10 and 5 min each time, respectively, and controlled central venous pressure below 4 mmHg during parenchyma dissection to control intraoperative bleeding. Complete hepatic resection was defined as the complete removal of all detected tumors without involving any major branch of the portal or hepatic veins, without invasion of adjacent organs and without lymph node or distant metastasis, and tumor-free margins confirmed by histopathology.

### Propensity Score Matching (PSM)

A PSM method was used to balance the potential biases between two groups. The propensity score was estimated using a multivariate logistic regression by using variables of diameter of largest HCC tumor, number of tumors, serum AFP level, microvascular invasion, tumor encapsulation, resection margin, and type of hepatectomy. Patients were matched 1:1 using the nearest neighbor method with a caliber of 0.05; the matching process has been described in a previous study (24).

### Follow-Up

The follow-up period for this study was terminated on September 30, 2019. Patients were followed at least once every 3 months after LR; the visits involved checking serum AFP levels and performing screening abdominal imaging (e.g., abdominal CT and/or MRI and/or ultrasound scans). HCC recurrence was suspected when there was a progressive elevation of serum AFP levels, a new showing contrast enhancement in the arterial phase and washout in the venous phase on CT and/or MRI, and/or hypervascularity on hepatic angiography.

The dates of tumor recurrence, last follow-up, and death were recorded. The primary endpoint was overall survival (OS), and the secondary endpoint was recurrence-free survival (RFS). OS was defined as the time from LR to death or last follow-up, and RFS was defined as the time from LR to tumor progression, death, or last follow-up (whichever came first). Tumor progression was defined as the local tumor recurrence or the occurrence of new lesions in the liver or elsewhere, based on imaging.

### Statistical Methods

For the study, the patients were divided into 2 groups, with those having TACE prior to LR placed in the TACE+LR group, and those having only LR placed in the LR group. The demographic and clinicopathological characteristics of the groups were summarized using frequencies and percentages for categorical covariates and means and standard deviation (SD) for continuous covariates. The Fisher exact test was used to compare categorical covariates, while the Wilcoxon rank-sum test was used to compare continuous covariates. The cutoffs for continuous variables were chosen to allow for easy interpretation. OS and RFS rates were calculated using the Kaplan–Meier method. Univariate and multivariate Cox regression analyses were used to determine the impact of risk factors on recurrence (using RFS) and death (using OS). Variables with *P*-values less than 0.10 in the univariate analysis were subjected to the multivariate Cox regression model using a forward stepwise variable selection; results were reported as hazards ratios (HR) and 95% confidence intervals (CI). A 2-tailed *P*-value less than 0.05 was considered statistically significant for all of the tests. Statistical analyses were performed using SPSS version 25.0 (IBM).

## Results

### Demographic and Clinicopathological Characteristics

A total of 488 patients met the inclusion criteria for the study. The mean follow-up period was 40.2 (range, 3.0 to 128.7) months. Of these, 223 (45.7%) were in the TACE+LR group and 265 (54.3%) were in the LR group (**Table 1**). When compared to the patients in the LR group, significantly more of those in the TACE+LR group had resection margins of 1 cm or less (89.7% vs. 76.6%, *P* < 0.001) and tumor encapsulation (70.0% vs. 60.8%, *P* = 0.03), and significantly less had microvascular invasion (27.4% vs. 51.5%, *P* = 0.001). Conversely, there were no significant differences between the 2 groups with regards to sex, age, BMI, tumor size, number of tumors, hepatitis, portal hypertension, comorbidity, AFP and all other biochemical blood tests, type of hepatectomy, cirrhosis, intraoperative blood loss and transfusion, and postoperative complications. A PSM model was established to balance the bias of clinicopathological characteristics between the two groups. As shown in **Table 1**, total of 378 patients were enrolled and 189 in each group. Resection margins, tumor encapsulation, and microvascular invasion presented no difference between the two groups after PSM, and other characteristics also showed no significance.

**Table 1:** Demographic and clinicopathological characteristics of patients with BCLC stage B hepatocellular carcinoma (HCC) before and after propensity score matching (PSM), by treatment (TACE+LR vs. LR), January 2007 to December 2015.

Characteristics	Before PSM		P	After PSM		P
	TACE+LRN (%)	LRN (%)		TACE+LRN (%)	LRN (%)	
Total patients	223 (100)	265 (100)	*-*	189	189	
Sex						
*Male*	197 (88.3)	238 (89.8)	0.60	169(89.4)	171(90.5)	0.73
*Female*	26 (12.7)	27 (10.2)	20(10.6)	18(9.5)		
Age, *years*						
*<60*	179 (80.2)	215 (81.3)	0.81	150(79.4)	155(82)	0.515
*≥60*	44 (19.8)	50 (18.7)	39(20.6)	34(28)		
Largest HCC tumor diameter, *cm*						
*<10*	150 (67.2)	199 (75.1)	0.056	137(72.5)	133(70.4)	0.65
*≥10*	73 (28.2)	66 (24.9)	52(37.5)	56(29.6)		
HCC tumors, *n*						
*≤3*	172 (77.1)	193 (72.8)	0.28	141(74.6)	143(75.7)	0.81
*>3*	51 (22.9)	72 (27.2)	48(25.4)	46(24.3)		
Hepatitis^a^	174 (78.0)	203 (76.6)	0.71	142(75.1)	147(77.8)	0.51
Portal hypertension^b^	18 (8.1)	11 (4.2)	0.07	12(6.3)	8(4.2)	0.36
Comorbidity^c^	25 (11.2)	27 (10.2)	0.72	21(11.1)	18(9.5)	0.61
Alpha-fetoprotein (AFP) level, *ng/ml*						
*≤400*	127 (57.0)	134 (50.6)	0.16	101(53.4)	103(54.5)	0.84
*>400*	96 (43.0)	131 (49.4)	88(46.6)	86(55.5)		
Type of hepatectomy^d^						
*Anatomical*	64 (28.7)	79 (35.4)	0.79	53(28)	60(31.7)	0.43
*Non-anatomical*	159 (71.3)	186 (64.6)	136(72)	129(68.3)		
Resection margin^e^, *cm*						
*≤1*	200 (89.7)	203 (76.6)	**<0.001**	166(87.8)	164(86.8)	0.76
*>1*	23 (10.3)	62 (24.3)	23(12.2)	25(13.2)		
Tumor encapsulation^f^	156 (70.0)	161 (60.8)	**0.03**	122(64.6)	119(63)	0.75
Microvascular invasion^g^	61 (27.4)	110 (41.5)	**0.001**	61(32.3)	59(31.2)	0.83
Cirrhosis^g^	117(52.5)	147(55.5)	0.507	107(56.6)	116(61.4)	0.347
Postoperative complication^h^	13 (6.0)	21 (8.0)	0.37	11(6)	16(8.5)	0.32
*Characteristics*	**Before PSM**		***P***	**After PSM**		***P***
	**TACE+LR** **Mean ± SD**	**LR** **Mean ± SD**		**TACE+LR** **Mean ± SD**	**LR** **Mean ± SD**	
Body Mass Index (BMI), *kg/m^2^*	22.2 ± 3.1	22.4 ± 3.1	0.61	22.2 ± 3.1	22.1 ± 2.7	0.65
Alanine aminotransferase (ALT), *IU/L*	56.1 ± 54.9	53.9 ± 49.4	0.63	58.7 ± 58.4	57.4 ± 55.2	0.65
Albumin (ALB), *g/L*	39.9 ± 6	40.7 ± 4.5	0.083	39.9 ± 6.5	40.6 ± 4.7	0.27
Total bilirubin (TBIL), *μmol/L*	13.8 ± 6.7	14.5 ± 6.5	0.22	13.9 ± 7.1	13.9 ± 5.4	0.98
White blood count (WBC), *x 10^9^/L*	6.4 ± 2.0	6.8 ± 3.1	0.08	6.4 ± 1.9	6.8 ± 3.2	0.075
Platelet count (PLT), *x 10^9^/L*	194.2 ± 85.1	195.9 ± 76.3	0.82	196.3 ± 84.4	200.3 ± 77.8	0.63
Prothrombin time (PT), *seconds*	12.1 ± 1.1	12.2 ± 1.1	0.43	12.1 ± 1.1	12.2 ± 1.1	0.74
International normalized ratio (INR)	1.1 ± 0.1	1.1 ± 0.1	0.70	1.1 ± 0.1	1.1 ± 0.1	0.51
Intraoperative blood loss, *mL*	651.6 ± 639.8	567.0 ± 794.2	0.20	673.3 ± 677.1	571.2 ± 637.2	0.13
Intraoperative blood transfusion, *mL*	176.5 ± 356.2	148.7 ± 378.8	0.41	177 ± 359.5	156.6 ± 379.3	0.59

a Hepatitis defined preoperatively as a history of chronic HBV infection and/or positive hepatitis C virus RNA test.

b Portal hypertension defined preoperatively as esophageal varices and/or splenomegaly on imaging studies combined with a decreased platelet count [100 × 10^3^/μL or less]).

c Comorbidity defined as preoperative hypertension, diabetes, coronary disease, and/or severe anemia.

d Determined by surgeon intraoperatively, anatomical approach based on the Brisbane 2000 nomenclature of liver anatomy, whereas non-anatomical approach consisted of wedge or limited resection.

e Based on intraoperative surgeon estimation.

f Based on intraoperative surgeon description of tumor appearing encapsulated or infiltrating (not encapsulated).

g Based on postoperative histopathology report.

h Postoperative large pleural effusion, pneumonia, portal vein thrombosis, and/or cholestasis.

BCLC, Barcelona Clinic Liver Cancer; TACE, transarterial chemoembolization; LR, liver resection (hepatectomy). All bold P value were represented the significance (P < 0.05).

### Overall Survival (OS)

**Figure 1:**
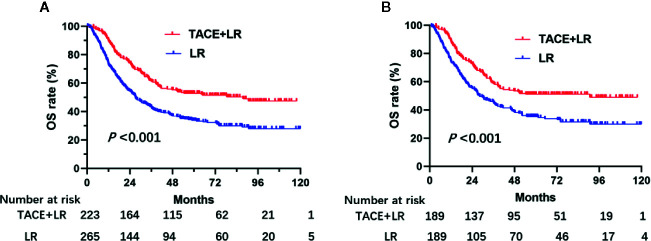
Kaplan–Meier overall survival (OS) rate curves for patients underwent TACE+LR and LR for BCLC stage B hepatocellular carcinoma (HCC) before and after PSM, January 2007 to December 2015. **(A)** the OS rate of patients before PSM, **(B)** the OS rate of patients after PSM. OS rates of the patients who received TACE+LR were significantly higher than OS rates of those who received only LR both before and after PSM (*P* < 0.001).

By the last follow-up, 107 (48%) patients in the TACE+LR group and 181 (68.3%) patients in the LR group had died. The 90-day mortality rate for patients in the TACE+LR group was 1.3% (3 patients) and for patients in the LR group was 4.2% (11 patients). Over the entire study period, patients in the TACE+LR group had significantly higher OS than patients in the LR group before PSM (*P* < 0.001) (**Figure 1A**). After PSM, the OS curve of patients in TACE+LR group showed better survival rate than LR group (*P* < 0.001) (**Figure 1B**). The estimated 1-, 3-, and 5-year OS rates for patients receiving TACE+LR were 90.6%, 61.7%, and 52.9%, respectively, whereas the rates for those receiving LR were 73.3%, 43.5%, and 33.8%, respectively (all *P* < 0.001) (**Table 2**).

**Table 2:** Overall survival (OS) and recurrence-free survival (RFS) rates in 488 patients with BCLC stage B hepatocellular carcinoma (HCC), by treatment (TACE+LR vs. LR), January 2007 to December 2015.

Rates**	Treatment		*P*
	TACE+LR (n = 223) %	LR (n = 265) %	
1-year OS	90.6	73.3	**<0.001**
3-year OS	61.7	43.5	**<0.001**
5-year OS	52.9	33.8	**<0.001**
1-year RFS	54.6	39.4	**<0.001**
2-year RFS	41.4	29.4	**0.002**
3-year RFS	36.3	26.3	**0.008**

BCLC, Barcelona Clinic Liver Cancer; TACE, transarterial chemoembolization; LR, liver resection (hepatectomy). Bold values provided in this table was that P value < 0.05, there was significant difference between the two groups.

Based on multivariate analysis, LR (vs. TACE+LR) as treatment (HR=1.94, 95% CI 1.52–2.48, *P* < 0.001), more than 3 (vs. 3 or fewer) tumors (HR=2.19, 95% CI 1.69–2.84, *P* < 0.001), non-anatomical (vs. anatomical) hepatectomy (HR=1.29, 95% CI 1.01–1.66, *P* = 0.046), microscopic vascular invasion (HR=1.46, 95% CI 1.15–1.90, *P* = 0.002), cirrhosis (HR=2.41, 95% CI 1.88–3.01, *P* < 0.001), and intraoperative blood transfusion (HR=1.29, 95% CI 1.01–1.66, *P* = 0.004) were all significantly independently associated with OS (**Table 3**).

**Table 3:** Univariate and multivariate analyses of demographic and clinicopathological prognostic factors for overall survival (OS) in 488 patients with BCLC stage B hepatocellular carcinoma (HCC), January 2007 to December 2015.

Characteristics	Variables	Univariate Analysis		Multivariate Analysis	
		Hazard Ratio (95% CI)	*P*	Hazard Ratio (95% CI)	*P*
Type of treatment	LR vs. TACE+LR	1.80 (1.42–2.29)	**<0.001**	1.94 (1.52–2.48)	**<0.001**
Age, *years*	>60 vs. ≤60	1.00 (0.75–1.34)	0.99	–	–
Sex	Male vs. female	0.94 (0.64–1.37)	0.73	–	–
Largest HCC tumor diameter, *cm*	≥10 vs. < 10	1.32 (1.03–1.7)	0.03	NS	0.30
HCC tumors, *n*	>3 vs. ≤3	2.52 (1.97–3.23)	**<0.001**	2.19 (1.69–2.84)	**<0.001**
Portal hypertension^a^	Yes vs. no	0.97 (0.60–1.59)	0.91	–	–
Alpha-fetoprotein (AFP), *ng/mL*	>400 vs. ≤400	1.46 (1.16–1.84)	**0.001**	NS	0.15
Type of hepatectomy^b^	Non-anatomical vs. anatomical	1.35 (1.05–1.73)	**0.02**	1.29 (1.01–1.66)	**0.046**
Resection margin^c^, *cm*	>1 vs. ≤1	1.09 (0.81–1.47)	0.56	–	–
Tumor encapsulation^d^	Yes vs. no	0.84 (0.66–1.06)	0.15	–	–
Microscopic vascular invasion^e^	Yes vs. no	1.94 (1.53–2.45)	**<0.001**	1.48 (1.15–1.90)	**0.002**
Cirrhosis^e^	Yes vs. no	2.67 (2.09–3.41)	**<0.001**	2.41 (1.88–3.01)	**<0.001**
Intraoperative blood transfusion	Yes vs. no	1.71 (1.33–2.20)	**<0.001**	1.45 (1.13–1.93)	**0.004**

a Portal hypertension defined preoperatively as esophageal varices and/or splenomegaly on imaging studies combined with a decreased platelet count [100 × 10^3^/μL or less]).

b Determined by surgeon intraoperatively, anatomical approach based on the Brisbane 2000 nomenclature of liver anatomy, whereas non-anatomical approach consisted of wedge or limited resection.

c Based on intraoperative surgeon estimation.

d Based on intraoperative surgeon description of tumor appearing encapsulated or infiltrating (not encapsulated).

e Based on postoperative histopathology report.

BCLC, Barcelona Clinic Liver Cancer; CI, confidence interval; TACE, transarterial chemoembolization; LR, liver resection (hepatectomy); NS, not significant. Bold values provided in this table was that P value < 0.05, there was significant difference between the two groups.

### Recurrence-Free Survival (RFS)

**Figure 2:**
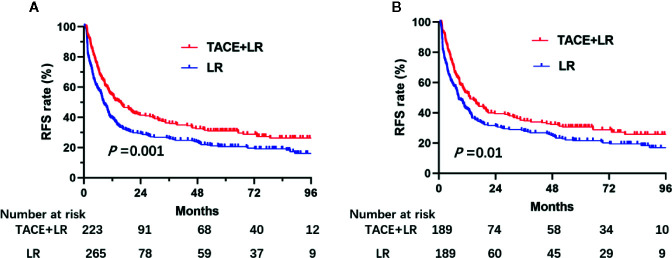
Kaplan–Meier recurrence-free survival (RFS) rate curves for patients underwent TACE+LR and LR for BCLC stage B hepatocellular carcinoma (HCC) before and after PSM, January 2007 to December 2015. **(A)** the RFS rate of patients before PSM, **(B)** the RFS rate of patients after PSM. Patients who received TACE+LR were significantly higher than RFS rates of those who received only LR both before and after PSM (*P* < 0.05).

By the last follow-up, 158 (70.9%) patients in the TACE+LR group and 213 (80.4%) patients in the LR group had experienced recurrence. Over the entire study period, patients in the TACE+LR group had significantly higher RFS than patients in the LR group (*P* = 0.001) (**Figure 2A**). After PSM, patients in TACE+LR group had obvious longer RFS than the LR group (*P* = 0.01) (**Figure 2B**). The median RFS in the TACE+LR group (15.4 months, 95% CI 10.2–20.6 months) was 7 months longer than in the LR group (8.3 months, 95% CI 8.6–12.2 months). The estimated 1-, 2-, and 3-year RFS rates for patients receiving TACE+LR were 54.6%, 41.4%, and 36.3%, respectively, whereas the rates for those receiving LR were 39.4%, 29.4%, and 26.3%, respectively (*P* < 0.001, *P* = 0.002, and *P* = 0.008, respectively) (**Table 2**).

Based on multivariate analysis, LR (vs. TACE+LR) as treatment (HR=1.55, 95% CI 1.26–1.91, *P* < 0.001), tumor size of 10 cm or more (vs. less than 10 cm) (HR=1.39, 95% CI 1.11–1.75, *P* = 0.005), more than 3 (vs. 3 or fewer) tumors (HR=2.98, 95% CI 2.35–3.79, *P* < 0.001), microscopic vascular invasion (HR=1.45, 95% CI 1.16–1.81, *P* = 0.001), and cirrhosis (HR=1.74, 95% CI 1.41–2.15, *P* < 0.001) were all significant independent predictors of recurrence (**Table 4**).

**Table 4:** Univariate and multivariate analyses of demographic and clinicopathological prognostic factors for recurrence-free survival in 488 patients with BCLC stage B hepatocellular carcinoma (HCC), January 2007 to December 2015.

Characteristics	Variables	Univariate Analysis		Multivariate Analysis	
		Hazard Ratio (95% CI)	*P*	Hazard Ratio (95% CI)	*P*
Type of treatment	LR vs TACE+LR	1.44 (1.17–1.76)	**0.001**	1.55 (1.26–1.91)	**<0.001**
Age, *years*	>60 vs. ≤60	0.97 (0.75–1.25)	0.80	–	–
Sex	Male vs. female	0.85 (0.60–1.20)	0.85	–	–
Largest HCC tumor diameter, *cm*	≥10 vs. < 10	1.51 (1.21–1.88)	**<0.001**	1.39 (1.11–1.75)	**0.005**
HCC tumors, *n*	>3 vs. ≤3	3.43 (2.73–4.32)	**<0.001**	2.98 (2.35–3.79)	**<0.001**
Portal hypertension^a^	Yes vs. no	1.00 (0.66–1.53)	0.99	–	–
Alpha-fetoprotein (AFP), *ng/mL*	>400 vs. ≤400	1.35 (1.10–1.65)	**0.004**	NS	0.12
Type of hepatectomy^b^	Non-anatomical vs. anatomical	1.31 (1.05–1.64)	**0.02**	NS	0.09
Resection margin^c^, *cm*	>1 vs. ≤1	0.97 (0.74–1.27)	0.83	–	–
Tumor encapsulation^d^	Yes vs. no	0.86 (0.70–1.07)	0.18	–	–
Microscopic vascular Invasion^e^	Yes vs. no	1.79 (1.45–2.21)	**<0.001**	1.45 (1.16–1.81)	**0.001**
Cirrhosis^e^	Yes vs. no	1.91 (1.55–2.36)	**<0.001**	1.74 (1.41–2.15)	**<0.001**
Intraoperative blood transfusion	Yes vs. no	0.92 (0.74–1.27)	0.83	–	–

^a^Portal hypertension defined preoperatively as esophageal varices and/or splenomegaly on imaging studies combined with a decreased platelet count [100 × 10^3^/μL or less]).

^b^Determined by surgeon intraoperatively, anatomical approach based on the Brisbane 2000 nomenclature of liver anatomy, whereas non-anatomical approach consisted of wedge or limited resection.

^c^Based on intraoperative surgeon estimation.

^d^Based on intraoperative surgeon description of tumor appearing encapsulated or infiltrating (not encapsulated).

^e^Based on postoperative histopathology report.

BCLC, Barcelona Clinic Liver Cancer; CI, confidence interval; TACE, transarterial chemoembolization; LR, liver resection (hepatectomy); NS, not significant. Bold values provided in this table was that P value < 0.05, there was significant difference between the two groups.

## Discussion

Patients with intermediate-stage HCC have large and multifocal HCCs and do not have evidence of intrahepatic macrovascular invasion or extrahepatic metastases (25). Progression after treatment continues to be a substantial challenge in the clinical management of patients with large HCC and is associated with poor survival outcomes. Currently, the most common treatment for intermediate-stage HCC is TACE (26). TACE concludes with selective embolization of HCC tumors. However, before that, the procedure involves the intra-arterial infusion of a chemotherapy agent embedded in lipiodol, which tends to accumulate in the blood and lymph vessels of tumors, and serves as a vehicle for prolonging tumor exposure to the agent, yet does not adversely affect normal liver cells (8) (27).

Some researchers suggest that the evidence supporting TACE as first-line treatment for intermediate-stage HCC may not be strong enough, and they suggest that because LR may result in better outcomes than TACE, it should be considered first-line treatment for most patients with intermediate-stage disease (28). Others have echoed this, suggesting that treatments more aggressive than TACE, such as LR or energy ablation, should be considered first-line treatment for intermediate-stage HCC (29, 30). Some may argue that the high number and large size of tumors in some patients with intermediate-stage HCC make LR an inferior option. However, several large studies have demonstrated that the number and size of HCC tumors should not be used as a selection criterion for LR, provided that tumor location and liver function would otherwise allow resection (31–34). The results of these studies suggest that patients with multiple HCCs and Child-Pugh classes A or B should be considered for LR. Furthermore, recent advances in surgical technique, perioperative care, and accurate patient selection have gradually reduced the morbidity and mortality of LR, and encouraging postoperative results and oncological outcomes are being reported in patients with intermediate-stage HCC (10, 35, 36).

In this retrospective clinical study, we looked not only at 256 patients who had received LR for intermediate-stage HCC but also at 223 patients who underwent LR preceded by TACE, over a 9-year period, with a mean duration of follow-up of 40.2 months. When we compared the 2 groups in the study, we found them to be well-matched, with no significant differences in demographic or preoperative clinical characteristics (including tumor size or number, as well as baseline hepatitis, comorbidity, or AFP levels), or in type of hepatectomy performed, histopathological evidence of cirrhosis, or postoperative complications.

However, we did find that relative to the group that underwent LR alone, a significantly higher proportion of patients in the group that received TACE+LR had intraoperative findings of narrow resection margins, and a significantly lower proportion in that group had postoperative histopathological evidence of microvascular invasion. These observations suggest that by exposing the disease to cytotoxic agents and then blocking tumor vessels, TACE may have created a strong cytotoxic effect and caused substantial tumor necrosis prior to surgery, resulting in tumor contraction, narrower margins, and eradication of some of the microvascular invasion. These findings and potential mechanisms are consistent with those reported by others (24). They conflict with a 1995 study from Wu et al., which suggested that TACE should be avoided prior to LR because it did not provide complete necrosis in large tumors (though it did result in a mean 42.8% reduction in tumor volume) and it resulted in delayed surgery (37). However, their study differed from ours in that most of their patients in the TACE+LR group had multiple TACE treatments, administered every 4 to 6 weeks, and the overall survival for their patients who received TACE+LR was worse than for those who received only LR.

When we compared survival outcomes, patients having TACE+LR showed significantly longer OS and RFS than those having only LR. For example, the 5-year OS rate for the TACE+LR group was 52.9%, whereas that for the LR group was 33.8%. Likewise, the 3-year RFS rate for the TACE+LR group was 36.3%, whereas that for the LR group was 26.3%. Our OS and RFS results for patients having TACE+LR were markedly better than those reported by Zhao et al. for patients with intermediate-stage HCC having TACE alone. The 5-year OS rate was 12% and the 3-year RFS rate was 25% for the patients treated with only TACE, despite the vast majority of their patients having only a solitary tumor (8). Taken together, these observations and our results suggest that the combination of TACE and LR could offer more effective tumor eradication than either TACE or LR alone for patients with intermediate-stage HCC, particularly among those with multifocal HCC.

As noted above, some authors in the past have suggested that doing TACE before LR may increase the risk of perioperative morbidity. However, in our study there was no significant difference in the proportion of patients having postoperative complications, when comparing those having LR with those having TACE before LR. Similarly, Li et al. showed not only that the addition of preoperative TACE to LR for huge HCC (10 cm or larger) was associated with an improved OS and RFS, but also that this combination therapy did not increase perioperative morbidity or mortality (24).

Compared to the group of patients in our study who had LR alone, those who received TACE before LR exhibited higher numerical mean volumes of intraoperative blood loss (652 mL vs. 567 mL) and intraoperative blood transfusion (177 mL vs. 149 mL), but these differences were not statistically significant. Some have suggested that patients with hepatitis who undergo preoperative TACE before LR might suffer more intraoperative bleeding and present more operative challenges than those who do not have TACE before LR (38). In our study, the proportion of patients with hepatitis in each group was not significantly different. This, combined with a lack of significant differences in blood loss and transfusion, may provide additional evidence that cirrhosis should not be considered a contraindication to TACE before LR. Some authors have reported that TACE had little influence on subsequent surgery if the interval between the last TACE and LR was long enough (39). The patients in our study who received preoperative TACE had an interval between the last TACE and LR of at least 4 weeks; this suggests the possibility that waiting at least 4 weeks between TACE and LR may result in a risk for bleeding and a need for transfusion that is closer to the risks for LR alone. Finally, the results of our study may provide some support for the proposal that the amount of intraoperative blood loss and blood transfusion during LR may more likely be a function of tumor size (40). The TACE+LR group in our study, which had numerically higher volumes of blood loss and transfusion, also had a higher proportion of patients with huge (10 cm or greater) HCC when compared to the LR group (28.2% vs. 24.9%, respectively), though once again these differences were not statistically significant.

On multivariate analyses, confirming our results based on Kaplan-Meier estimates, we observed that LR alone was independently associated with HCC recurrence and death. Furthermore, we observed that more than 3 HCC tumors, non-anatomical hepatectomy, microscopic vascular invasion, cirrhosis, and intraoperative blood transfusion were all independent predictors of poor OS. Most of these results echo the findings from several other studies of patients who have had LR for intermediate-stage HCC (1, 25, 41). However, the influence of intraoperative blood transfusion on post-LR outcomes continues to be debated. Several studies have observed that intraoperative transfusion had no influence on the OS of patients with HCC after LR (1, 24, 41). In contrast, Mori et al. reported that perioperative blood transfusion was an independent risk factor for poor prognosis after curative surgery for primary HCC in a multi−center study (42). Similarly, Maehara et al. reported that the presence of intraoperative transfusion was an independent poor prognostic factor for OS in patients having LR for HCC of 5 cm or larger (16). Our findings are in line with the studies from Mori et al. and Maehara et al.

This study has several limitations. First, this was a retrospective, single-center study that lacked randomization. The fact that the choice of treatment was made at the discretion of the treating physician may have introduced selection bias. Second, this study does not address which patients might benefit most from the addition of TACE prior to LR. It would be worthwhile to conduct an additional study to develop clinical prediction models (including with the possible use of radiomics) to identify the population that would be best served by the addition of TACE to LR for intermediate-stage HCC.

## Conclusion

In this large retrospective study of patients with intermediate-stage HCC, the addition of TACE 1 to 3 months prior to LR resulted in significantly longer OS and RFS compared to LR alone. These results and comparisons with findings from other studies suggest that preoperative TACE with LR may result in better oncological outcomes than either TACE or LR alone, without a substantial increase in morbidity, and that this approach could be considered an effective combination treatment for intermediate-stage HCC.

## Data Availability Statement

All datasets presented in this study are included in the article/supplementary material.

## Ethics Statement

The studies involving human participants were reviewed and approved by Ethics Committee of the Sun Yat-sen University Cancer Center. The patients/participants provided their written informed consent to participate in this study.

## Author Contributions 

Conceptualization: MC, JH. Data curation: QZ, JW, ZH, FT. Formal analysis: QZ, XW, RL. Data analysis: RL, QZ. Funding acquisition: JH, QZ. Investigation: QZ, JW, ZH. Methodology: QZ, XW, ZH. Project administration: QZ, XW, JH, MC. Resources: QZ, MC, JH. Original draft: QZ, XW. Writing—review and editing: QZ, MC, JH. All authors contributed to the article and approved the submitted version.

## Funding

This research was funded by the Chinese Postdoctoral Science Foundation (No. 2019M653203); National Natural Science Foundation of China (No. 81771955).

## Conflict of Interest

The authors declare that the research was conducted in the absence of any commercial or financial relationships that could be construed as a potential conflict of interest.
